# Complications of Capsulectomies: An Analysis of the American College of Surgeons National Surgical Quality Improvement Program Database

**DOI:** 10.1093/asjof/ojac025

**Published:** 2022-04-12

**Authors:** Jad Abi-Rafeh, Tyler Safran, Sebastian Winocour, Tassos Dionisopoulos, Peter Davison, Joshua Vorstenbosch

**Affiliations:** Division of Plastic and Reconstructive Surgery, McGill University Health Center, Montreal, QC, Canada; Division of Plastic and Reconstructive Surgery, McGill University Health Center, Montreal, QC, Canada; Division of Plastic and Reconstructive Surgery, Baylor College of Medicine, Houston, TX, USA; Division of Plastic and Reconstructive Surgery, McGill University Health Center, Montreal, QC, Canada; Division of Plastic and Reconstructive Surgery, McGill University Health Center, Montreal, QC, Canada; Division of Plastic and Reconstructive Surgery, McGill University Health Center, Montreal, QC, Canada

## Abstract

**Background:**

Although plastic surgeons commonly perform capsulectomies for a variety of peri-prosthetic capsular conditions, the safety of capsulectomy remains unknown, and the literature lacks evidence describing its morbidity and complication rates for patients inquiring about its associated risks.

**Objectives:**

The present study aims to identify and define the complication rates associated with capsulectomies.

**Methods:**

An analysis of the American College of Surgeons National Surgical Quality Improvement Program (NSQIP) database was performed between the years 2015 and 2018. All information pertaining to demographics, patient-related information, surgical indications, procedure-related information, outcomes, and complications were assessed.

**Results:**

The study identified 2231 cases of surgeon-reported capsulectomies; indications most commonly reported included capsular contracture (n = 638, 28.6%) and breast implant rupture (n = 403, 18.1%). In total, 141 patients (6.32%) were hospitalized for longer than 1 postoperative day (range, 2-28 days), while the overall complication rate was 3.0% (n = 67/2231 patients). Incidence of minor complications, representing superficial surgical site infections, was 0.8%, while the major complication rate was 2.24%. These included 7 cases of deep surgical site infections (0.3%), 19 organ space infections (0.9%), and 8 cases of wound dehiscence (0.4%). Eight patients developed sepsis (0.4%); 6 patients required transfusions (0.3%); 1 case of postoperative pneumonia and 1 myocardial infarction were also identified (n = 1 each, 0.0%). The overall reoperation and readmission rates were 2.0%, representing a readmission rate of 66% among patients with complications.

**Conclusions:**

The present study provides the first estimate of the incidence of complications associated with capsulectomies. Although the NSQIP database contains significant limitations, the data presented herein describe a complication profile that plastic surgeons can share with their patients during informed consent.

**Level of Evidence: 4:**

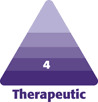

See the Commentary on this article here.

Reconstructive and aesthetic breast implant surgery has experienced a notable increase in popularity over the past decade^1,2^; each year, it is estimated that over 1.5 million breast implants are used for both reconstructive and aesthetic purposes, respectively.^3,4^ However, up to 19% of patients with breast implants, and as high as 40% of implanted patients with a previous history of breast irradiation are estimated to develop capsular contracture.^5^ Surgical management of capsular contracture varies but may comprise of either capsulotomies or capsulectomies depending on severity.^5,6^ Capsulectomies have thus come to represent a procedure commonly performed by plastic surgeons worldwide in remedy of not only capsular contracture,^6^ but also implant rupture,^7^ and more recently, breast implant-associated anaplastic large cell lymphoma (BIA-ALCL)^8^ and breast implant illness (BII).^9^ In recent years, patient-driven requests for complete intact capsulectomies have also been on the increase for prophylaxis against BIA-ALCL and BII, although the evidence for their efficacy in this context, specifically in asymptomatic patient cohorts, remains elusive.

A study performed previously by our group demonstrated that, despite the growing popularity of capsulectomy, the literature remains devoid of evidence on its complication rate profile.^10^ Given its growing popularity, it remains imperative to characterize and define its complications, according to its different subtypes, and associated clinical indications. The National Surgical Quality Improvement Quality Program (NSQIP) database represents a reliable, risk-adjusted, and case-mix-adjusted dataset established by the American College of Surgeons to facilitate access to data across participating institutions, with the goal of catalyzing improvements in patient care, morbidity, and cost savings.^11-13^ Within the plastic surgery literature, in particular, the NSQIP database has helped to characterize the incidence and define significant predictors of complications associated with an array of procedures, ranging from breast reconstruction, body contouring, to craniofacial surgery.^14-16^ In the present study, the authors perform an analysis of the NSQIP database in an effort to gain insight into the incidence and possible predictors of specific complications associated with different capsulectomies.

## METHODS

An analysis of the American College of Surgeons NSQIP database was performed using participant user file data between the years 2015 and 2018 in order to identify patients in whom capsulectomies were performed. Given the de-identified nature of the data in the NSQIP database, this study was deemed exempt from IRB review; written consent was provided by patients at American College of Surgeons NSQIP participating institutions, by which the patients agreed to the use and analysis of their data. Current Procedural Terminology (CPT) code 19371 was used to identify patients who had undergone complete capsulectomies. Data collection and analysis were performed by 2 independent evaluators. Patient-related data queried included age, body mass index, ethnicity, year of surgery, smoking status, history of alcohol use, American Society of Anesthesiologists (ASA) score, wound class, World Health Organization (WHO) obesity class, diabetes status, presence of hypertension, or previously diagnosed cardiovascular, pulmonary, neurological, renal, or hepatic conditions. Additionally, information relating to a history of chronic steroid use, recent weight loss, or a bleeding disorder was considered. Data relating to operative time, duration of hospitalization, and indications for surgery were also examined.

Outcomes of interest included all postoperative complications and adverse outcomes reported within the NSQIP database, including transfusion requirements, reoperation within 30 days, wound complications (superficial, deep, or organ space surgical site infections), sepsis or septic shock, postoperative pneumonia, unplanned intubations, pulmonary embolism, deep venous thrombosis, postoperative renal insufficiency, urinary tract infection, stroke, myocardial infarction, or death. Statistical analyses were performed using SPSS software.

## RESULTS

### Demographics and Patient Information

Our query of the NSQIP database identified 2231 patients reported to have underwent capsulectomies between the years 2015 and 2018. Patient demographics are presented in Table 1. The average computed BMI was 27.6 kg/m^2^, while information pertaining to patient ages was not available. We found 10.4% of patients to be smokers, 6.0% diabetic, and 0.2% reported to present with a 10% weight loss history in the past 6 months. Patient ASA classification was predominantly class 2 (65.8%), followed by class 3 (22.1%), class 1 (11.4%), and class 4 (0.6%). One percent of capsulectomies was reported to be performed on an “emergency basis,” while the vast majority of procedures were conducted under general anesthesia (99.4%). Wound class classification was reported to be predominantly clean (89.2%), followed by dirty/infected (5.1%), clean/contaminated (3.2%), and contaminated (2.5%; Table 1).

**Table 1.** Demographics and Patient-Related Information

Variable	Level	N = 2231
Patient-related information	Average height = 62.94 inch	—
	Average weight = 155.49 lb	—
	Average BMI = 27.6 kg/m^2^	—
	Smoker	231 (10.4%)
	Diabetic	134 (6.0%)
	>10% weight loss in last 6 months	5 (0.2%)
	Emergency case	23 (1.0%)
Patient ASA class	1	254 (11.4%)
	2	1467 (65.8%)
	3	493 (22.1%)
	4	14 (0.6%)
	5	0 (0%)
	n.s	3 (0.1%)
Wound class	1 (Clean)	1990 (89.2%)
	2 (Clean/Contaminated)	72 (3.2%)
	3 (Contaminated)	56 (2.5%)
	4 (Dirty/Infected)	113 (5.1%)
Principle anesthesia technique	General	2217 (99.4%)
	MAC/IV	10 (0.4%)
	Spinal, local, or epidural	4 (0.2%)

ASA, American Society of Anesthesiologists; IV, intravenous; MAC, monitored anesthesia care; ns, not specified.

### Indications for Capsulectomy and Procedure-Related Information

The most common capsulectomy indications were capsular contracture (n = 638, 28.6%), implant rupture (n = 403, 18.1%), and breast cancer or its recurrence (n = 195, 8.7%). Specific indications for capsulectomies were not specified in 407 cases (18.2%), although breast reconstruction following mastectomy (n = 162, 7.3%), breast/nipple absence or hypoplasia (n = 92, 4.1%), infection/ inflammation (n = 78, 3.5%), and implant-related pain/mastodynia (n = 73, 3.3%) were also described. Only 2 cases (0.1%) of capsulectomies were reported to be performed for the management of BIA-ALCL, and none were described to be performed specifically for BII. A detailed summary of all listed surgical indications is presented in Table 2. No information was available pertaining to whether capsulectomies performed were either complete or completely intact. Furthermore, no information was available relating to the plane of original implant insertion, implant texture, or previous history of irradiation.

**Table 2.** Indications for Surgery

Indication	Incidence
Capsular contracture and/or acquired breast deformity	638 (28.6%)
Mechanical implant complication/rupture	403 (18.1%)
Breast cancer and/or breast cancer recurrence	195 (8.7%)
“Breast reconstruction following mastectomy”	162 (7.3%)
Breast/nipple absence or hypoplasia	92 (4.1%)
Infection/inflammation	78 (3.5%)
Implant-related pain/mastodynia	73 (3.3%)
Implant fitting, adjustment, or explanation	67 (3.0%)
“Cosmetic surgery”	48(2.2)
Hematoma or seroma	24 (1.1%)
Implant displacement	18 (0.8%)
Ptosis/skin redundancy	11 (0.5%)
Disruption of surgical wound	7 (0.3%)
Fat necrosis/fibrosclerosis/skin fibrosis	6 (0.3%)
Anaplastic large cell lymphoma	2(0.1%)
Other/not specified	407 (18.2%)

### Operative Outcomes and Complications

Of the 2231 capsulectomy procedures reviewed, the average operative time was 102.28 minutes, while the average duration of hospitalization was 0.3 days. The latter ranged from 0 to 28 days, and 141 patients (6.32%) were hospitalized for longer than 1 day postoperatively. Among this cohort, 94 patients (67%) had at least one other concomitant procedure performed at the time of initial surgery. When analyzing CPT codes of concomitant procedures performed, 16.7% appeared to be relating to additional major breast surgery, such as free flap breast reconstruction (n = 4 cases, 1.9%). Additionally, 7.7% of concomitant procedures appeared to be performed for the management of a previous complication. (Table 3; Supplementary Table 1).

**Table 3.** Outcomes

Outcomes	Level	Incidence (n)
Average operative time = 102.28 min	—	—
Average duration of hospitalization = 0.3 days	—	—
Duration of hospitalization	0 day	1854 (83.1%)
	1 day	237 (10.6%)
	2-3 days	71 (3.2%)
	4-5 days	41 (1.9%)
	6-8 days	18 (0.8%)
	10-15 days	7 (0.3%)
	16-28 days	4 (0.0%)
Reoperation rate	—	44 (2.0%)
Readmission rate	—	44 (2.0%)
Reason for readmission	SSI Sepsis MI Pneumonia Other/not specified	15 2 1 1 25
Wound complications	Superficial SSI Deep SSI Organ space SSI Wound dehiscence	17 (0.8%) 7 (0.3%) 19 (0.9%) 8 (0.4%)
Sepsis	—	8 (0.4%)
Postoperative pneumonia, %	—	1 (0.0%)
Myocardial infarction, %	—	1 (0.0%)
Transfusion for intraoperative bleeding	—	6 (0.3%)
Days from operation until transfusion	0 day 1 day 3 days	2 3 1

MI, myocardial infarctions; SSI, surgical site infections.

The overall cumulative complication rate was 3.0% (n = 67/2231 patients). Complications comprised 17 cases of superficial surgical site infections (0.8%), 7 cases of deep surgical site infections (0.3%), 19 organ space surgical site infections (0.9%), and 8 cases of wound dehiscence (0.4%). Eight patients were reported to develop sepsis (0.4%); 6 patients required transfusions for intraoperative bleeding (0.3%), while 1 case of postoperative pneumonia and 1 myocardial infarction were also reported (n = 1 each, 0.0%). The overall reoperation and readmission rates were 2.0% (n = 44 patients). Reasons for readmission comprised surgical site infections (n = 15, 0.7%), sepsis (n = 2, 0.1%), myocardial infarction (n = 1, 0%), and pneumonia (n = 1, 0%). The specific reason for readmission was left unspecified in 25 cases (1.1%).

## DISCUSSION

Capsulectomies have come to represent a procedure commonly performed by plastic surgeons worldwide for the management of capsular contracture, breast implant rupture, and more recently, BIA-ALCL and BII.^3,4^ A previous study by our group demonstrated that, at present, there exist no data on the complication rate profile of capsulectomies that can serve to guide evidence-based management of these various conditions.^10^ Furthermore, paucity of the available evidence poses a challenge to the adequate assessment of the clinical indications for capsulectomies, and the risk-benefit considerations constantly deliberated by patients and healthcare professionals for interventions to manage these various conditions.^3,4^ To the authors’ knowledge, this is the first study of its kind to provide an estimate on the complication and reoperation rates of capsulectomy, which we report as 3.0% and 2.0%, respectively. However, entries retrieved from the NSQIP database remained devoid of insight into the specific plane of original implant insertion, a critical consideration with significant impact on outcomes. Therefore, while we provide some degree of insight into the morbidity of this procedure, there remains a need for further focused efforts, either prospective or retrospective in nature, that can serve to provide more detailed insight into the complication rate profile of this procedure in order to better inform clinical guidelines on optimal management strategies of various breast conditions.

### Nomenclature and Operative Techniques

Recent studies have drawn attention to the lack of consensus that exists within the nomenclature of capsulectomy, both from within the plastic surgery literature and among the general public, especially given growing concerns observed regarding BIA-ALCL and BII.^17^ Gerzenshtein^17^ clarifies that procedures termed as partial capsulectomies must represent the removal of only a problematic portion of a breast capsule when other sections may be either too thin or have no perceived benefit for removal.^17^ Complete capsulectomies would involve disruption and complete removal of the breast capsule in a manner that may culminate in the exposure of intracapsular contents to surrounding tissues, while intact complete capsulectomies represent complete removal of an intact breast capsule, along with its undisrupted intracapsular contents.^17^ The author clarifies that an *en bloc* capsulectomy is a procedure rarely truly performed, which, in its true surgical sense, would involve complete removal of an intact breast capsule with a defined margin of healthy issue on gross and histopathologic examination.^17^ Although growing in popularity among patients, and more commonly requested by the general public at what appears to be the suggestion of social media rather than clinical or scientific evidence,^10,18^ this procedure is presently reserved for severe cases of invasive BIA-ALCL, or invasive metaplastic processes of the breast capsule itself. Operative techniques thus differ according to not only the type of capsulectomy performed but also the plane of original implant insertion. Accordingly, it is postulated that capsulectomies performed within the submuscular plane are associated with greater morbidity, given that the anterior capsule is adherent to the well-vascularized *pectoralis* muscle and posterior capsule firmly adherent to the chest wall.^6^ Indeed, hematomas, pneumothoraxes, and injury to adjacent structures have all been widely observed, although the specific incidence of each, and within each capsulectomy type and the plane of implant insertion, remains elusive.^19,20^ Such challenges may be further exacerbated in the setting of post-mastectomy radiation therapy, even within the prepectoral plane, wherein simple capsulotomies may risk breaching thin fibrosed mastectomy skin flaps and culminate in adverse outcomes. In the present study, although a total of 2231 cases of surgeon-reported capsulectomies were identified from the NSQIP database, it remained unclear the specific types of capsulectomies performed. While the indication for capsulectomy was reported as BIA-ALCL in 2 cases, it may be presumed that en bloc capsulectomies were performed, and, by mere nature of the surgical procedure, as outlined above, these cases would have presented with greater risk for complications.

### Indications and Current Evidence

#### Capsular Contracture

At present, surgical management of significant capsular contracture revolves around either capsulotomy or capsulectomy, site change, and implant exchange, with an evolving role of acellular dermal matrix use to reduce the risk of recurrence.^6,21^ A recent systematic review by Wan and Rohrich^6^ demonstrated that currently, there exists limited consensus on optimal surgical management strategies of severe capsular contracture. Indeed, reported rates of recurrence remain widely variable between capsulectomy and capsulotomy cohorts, at 0%-46% vs 0%-54%, respectively.^6,22-30^ Furthermore, there exists significant variability in follow-up times and selection bias (predominantly prepectoral vs subpectoral contracture cohorts) across available studies, preventing adequate insight into the relative efficacy and recurrence rates between complete or partial capsulectomies.^29,30^ Therefore, at present, and although capsular contractures represented the most commonly encountered indication for capsulectomy in the NSQIP database (n = 638 patients, 28.6%), there exists limited evidence on the efficacy and clinical utility of complete capsulectomies for the management of capsular contracture, where the risks of both hematomas and penumothoracies may be significantly greater in the subpectoral space. Additionally, capsulectomies performed in irradiated, fibrotic breast tissue render the dissection more difficult and may predispose to more complications. Indeed, 6 patients within the cohort examined were reported to require transfusions for significant intraoperative bleeding, whereas 2% of all patients undergoing capsulectomy required readmission. Current recommendations thus suggest that total capsulectomies may be reserved for the prepectoral plane, and anterior, partial capsulectomies to the subpectoral space in order to limit injury to the chest wall.^6^ However, the choice of capsulectomy remains ultimately made on a patient-by-patient basis, and in consideration of the risks and potential benefits associated with each clinical scenario. Although an overall complication rate of 3.0% and total reoperation rate of 2.0% were established in the present study, limited information was specified within the NSQIP database entries, thus limiting further guidance in this context.

#### Breast Implant Rupture

Current literature concerning the management of silicone breast implant ruptures remains elusive. In the present study, 18.1% of patients receiving capsulectomies reported in the NSQIP database were as a result of breast implant rupture; however, no information was available regarding the intra- or extra-capsular nature of the ruptures examined, and their influence on complications observed. At present, recommendations regarding the management of symptomatic ruptures advocate for complete capsulectomies; however, debate persists regarding specific indications for partial or complete capsulectomies when ruptures remain asymptomatic.^31,32^ In the case of intracapsular ruptures, some surgeons advocate for leaving the prosthetic capsule intact and avoiding capsulectomies if its deemed intraoperatively that the silicone particulate matter has been adequately cleared and irrigated from within the intracapsular pocket.^31,33^ In contrast, if silicone gel remains within the capsule due to complicated ruptures, or the inability to adequately clear the pocket from debris, there would persist a risk of subsequent inflammation and calcification of the capsule that would warrant a complete capsulectomy.^7,31,33,34^ Nonetheless, current recommendations remain based on anecdotal evidence, and the literature remains devoid of a consensus on standardized, optimal management strategies for breast implant rupture. Information provided from the NSQIP database falls short of further clarifying this point.

#### BIA-ALCL and BII

BIA-ALCL and BII have raised significant safety concerns in recent years.^3,35^ As many as 1:354 patients with textured implants are reported to develop BIA-ALCL,^36^ and the National Comprehensive Cancer Network (NCCN) recommends complete intact capsulectomy as the treatment of choice in patients with confirmed disease.^3,35^ In asymptomatic patients with no indications of active disease, the FDA currently does not recommend implant exchange or capsulectomy.^10^ Despite these recommendations, there exists a growing population of patients presenting to plastic surgeons seeking complete capsulectomies on an elective basis due to their concern of developing BIA-ALCL or, as most recently noted, from fear of BII.^10,18^

BII was first described in the 1960s as a possible silicone adjuvant disease, encompassing a wide range of nonspecific clinical signs and symptoms.^3,9,18,37^ While the pathophysiology of BII remains unknown and subject to great controversy, recent studies have demonstrated clinically significant and sustained improvements in 11 common symptom domains within 30 days of explantation and complete capsulectomy.^37^ Debate persists on whether a capsulectomy is truly warranted in cases of BII, given that risks and complications associated with complete capsulectomy may outweigh its elusive benefits.^38^ As with patients seeking prophylactic capsulectomies from fear of BIA-ALCL, it remains critical to inform patients seeking capsulectomies for BII of its associated risks and complications during the informed consent process.

### Complications of Capsulectomy and NSQIP Limitations

Complications identified from within the NSQIP database with relevance to capsulectomies were predominantly infectious in nature. While superficial SSI and deep SSI may not have been exclusive to the procedure itself, “organ space infections” may have represented violations of the pleura that may have possibly culminated in empyemas; however, from the information gathered in the present study, this cannot be determined with certainty, and given the absence of clinical reports of empyema as a complication of capsulectomy, the clinical relevance of these particular outcomes remains inconclusive. Similarly, and while the reoperation rate of capsulectomies was determined to be 2.0%, close to half of these cases, which included SSIs, sepsis, MI, and pneumonia, also appeared to be not in strict relation to capsulectomies in question, with the remainder of reoperations remaining largely for unspecified causes. The 2 most critical outcomes identified from the available data that may contribute to the understanding of the procedure’s morbidity may be the duration of hospitalization and transfusion requirements. Hospitalizations greater than 1 day in duration may be indicative of adverse operative outcomes following capsulectomies. The cumulative incidence of admissions greater than 1 day, ranging from 2 to 28 days, was 6.2%. Although this value may too have been marginally overestimated by complications not necessarily related to capsulectomies, such as superficial surgical site infections; a large proportion may be indeed attributed to expected adverse outcomes such as hematomas, pneumothoracies, and inadvertent injury to adjacent structures within the plane of dissection arising as a result of more aggressive, or complete capsulectomies. Transfusion requirements for intraoperative bleeding had an incidence of 0.3%, which may provide further insight into the incidence of significant bleeding outcomes associated with likely more aggressive capsulectomies.

The use of the NSQIP database is thus not without its limitations. It remains unclear the clinical scenarios in which capsulectomies are performed, wherein wounds operated on would be classified as contaminated or infected. Additionally, an array of reported reasons for capsulectomy did not fit currently accepted indications, such as “breast cancer,” “breast/nipple absence or hypoplasia,” or even “ptosis/skin redundancy.” Other cases, such as “implant-related pain/ mastodynia,” “implant displacement,” or “implant fitting, adjustment, or explantation,” may have been performed for capsular contracture or BII, although this, too, remains inconclusive and speculative at best. Only 2 cases of BIA-ALCL were identified, and close to 18% of cases of capsulectomies did not have specified indications. It additionally remains unclear the proportion of observed complications arising as a result of concomitant procedures performed along with the capsulectomies examined, or whether surgeons performing the capsulectomies were board certified in plastic surgery. Finally, while data obtained from the NSQIP database provide a sense of the number of capsulectomies performed in the public setting at participating NSQIP institutions, the database fails in capturing the large volume of capsulectomies performed in private settings for complications of both aesthetic and reconstructive alloplastic breast procedures, including capsular contracture, breast implant rupture, and BIA-ALCL and BII. Nonetheless, and despite the aforementioned limitations, the present study provides, for the first time, insight into the complication rate profile and incidence associated with the procedure with the hope of catalyzing further research progress on this subject.

## CONCLUSIONS

The present study is the first of its kind in providing an estimate on the incidence of complications associated with capsulectomies, although NSQIP data used present with significant limitations. As capsulectomies continue to grow in popularity for the management of various evolving sequelae of alloplastic breast plastic surgery, there exists a marked indication for further clinical studies that can serve to elaborate on the present findings, with a focus on the complication rate profiles of different forms of capsulectomies, under different clinical indications, and within prepectoral and subpectoral planes, independently.

## Supplementary Material

ojac025_suppl_Supplementary_Table_S1
