# Supplementary material for: Complications of Capsulectomies: An Analysis of the American College of Surgeons National Surgical Quality Improvement Program Database
Source: Aesthet Surg J Open Forum. 2022 Apr 12;4:ojac025. doi: 10.1093/asjof/ojac025 (PMC9212085; doi:10.1093/asjof/ojac025)
Supplement: ojac025_suppl_Supplementary_Table_S1 [file ojac025_suppl_supplementary_table_s1.docx]

**Supplementary Table 1**. Concomitant Procedures in Patients with >1 Day Duration of Hospitalization

| Concomitant Procedures | Incidence |
| --- | --- |
| Minor  Explantation  Mammaplasty / Revision of Previous Reconstruction  Capsulectomy (other breast)  Fat Grafting / Adjunctive Tissue Transfer  Implant Placement / Implant Exchange  Expander Placement  Expander Implant Exchange  Capsulotomy  Anesthesia (Nerve Block) | 126 (60.2%)  32 (15.3%)  25 (12.0%)  19 (9.1%)  15 (7.2%)  12 (5.7%)  11 (5.3%)  8 (3.8%)  2 (1.0%)  2 (1.0%) |
| Major  Major Breast Surgery  IV Injection to Test Vascular Flow  Partial Rib Excision  Myocutaneous / Facsiocutaneous Flap (Trunk)  Free Flap  Lymph node Excision / Dissection / Biopsy  Breast Reconstruction (type unspecified)  Partial Mastectomy  Relating to Previous Complication  Deep Abscess / Complex Wound Irrigation and Debridement  Debridement  Hematoma or Seroma Irrigation and Debridement | 51 (24.4%)  35 (16.7%)  10 (4.8%)  9 (4.3%)  5 (2.4%)  4 (1.9%)  3 (1.4%)  3 (1.4%)  1 (0.5%)  16 (7.7%)  8 (3.8%)  6 (2.9%)  2 (1%) |
| Other / Unspecified in Detail | 32 (15.3%) |
| Total | 209 (100%) |
